# Observations on the Nature and Treatment of Spina Bifida

**Published:** 1840-08

**Authors:** Amasa Trowbridge

**Affiliations:** Professor of Surgery in Willoughby University, Painsville, Ohio


### WESTERN JOURNAL

OF

### MEDICINE AND SURGERY.

---

AUGUST, 1840.

---

ART. I.—*Observations on the Treatment of Spina Bifida.* By AMASA TROWBRIDGE, M.D., Professor of Surgery in Willoughby University, Ohio.

IN the Western Journal for May, I noticed some suggestions in relation to a new and different mode of treating Spina Bifida from those heretofore practised, which induce me to make this communication. Much observation and practice in this disease have convinced me, that it is important for surgeons to possess a correct pathological knowledge of the nature and formation of these tumours before they attempt their cure, or adopt any mode which may be suggested or practised for that object.

In 1829, I reported in the Boston Medical and Surgical Journal, (Vol. 1, No. 48,) three cases of spina bifida cured by operations. The cases with the modes of proceeding with each, were particularly detailed. In two of the cases, the tumours were removed by ligature, and in one by incision. I remarked in that communication, that I preferred the ligature in all cases where the base of the tumour admitted of its application—applied in the first place tight enough to produce moderate inflammation; after this the patient did not suffer as much in bringing it so tight, as to destroy all circulation, and its sympathetic effects were much diminished. I further remarked, that in making that communication public, I did not wish it to be understood, that I believed that all cases of spina bifida could be cured by any mode of treatment. I knew there were many that would soon destroy the child, and some that proved fatal even at their very birth, by being torn off in that process. Three cases of the latter I had witnessed. I also stated that I had seen about thirty cases, and had seen them situated upon every portion of the vertebral column, from the occipital bone to the sacrum, of various sizes and appearances, and had made many attempts to relieve the unhappy sufferers from their embarrassments, by puncture, compression, ligature, incision, et cætera, and that I had failed in my attempts in many instances; but that after all, I believed there were cases that could be cured, and that the surgeon, if he made the necessary distinction in the cases, might venture with as much assurance of success as he could in other maladies that required operations.

It is generally supposed that all cases are alike—that there is a communication from the sac, or outer cavity, through the spinal canal to the brain; but this is not the fact. There are cases where the opening through the vertebræ does not extend far into the canal, and where the spinal marrow is not compressed by fluid, or deranged in its functions; and many of these cases may be cured by surgical interference.

The external tumour in these cases, is generally covered entirely with integuments, and appears very firm, is not easily compressed, very elastic to the touch, and yet fluctuation is perceptible. In this case, the dura mater is the only lining membrane of the cavity in the tumour, and the fluid contained in this does not extend far in the canal after passing the aperture in the spinous apophyses. In the other cases, and these are much the most frequent, there is a thin, almost transparent membranous covering on many parts of the tumour, and sometimes on the greatest portion of it. The tumour is easily compressed, and the subject is generally attended with loss of voluntary action in some of the muscles, and sometimes with derangement in the secretions. Here the fluid is contained in the cavity of the arachnoid membrane, and may extend the whole length of the canal; and M. Bichat and other anatomists assert that it can extend even to the lateral ventricles of the brain. I have seen cases where the head was enlarged in a manner similar to what takes place in cases of hydrocephalus. These cases will soon prove fatal under any treatment that I have seen adopted. Since I reported the cases referred to in the Boston Journal, another has passed under my treatment, equally interesting, which goes to establish my views about these cases.

Miss M. Brayton of Jefferson county, New York, of a very respectable family, aged 25, was born with a tumour over the two lower lumbar vertebræ of considerable size and protuberance, which was pronounced by several medical gentlemen of eminence to be spina bifida. It was so large and so distinctly characterized at this time, that they predicted the child would live but a few weeks. She however continued to live in this state without much constitutional derangement or local suffering, except feebleness, during the first twelve years. The tumour enlarged in proportion to the growth of other parts of her body. From this period to the time when I first saw her, at the age of 25, she was occasionally troubled with extreme pain in the cerebral region of the head, and much disturbance of mind, attended with a vertigo and confusion of mental powers, and some neuralgic symptoms over the whole body. She was of the phlegmatic temperament and possessed a mind that was retiring and delicate. The secretions of the system were natural, and no delay was experienced in the usual infantile developments, either physical or mental. She was of the middling stature, and had the perfect use of all the muscles of the body. A family physician attended her during the paroxysms of pain and nervous excitement mentioned, but was ignorant of the real cause of these symptoms. He treated by bleeding, cathartics, opium, et cætera.

The tumour enlarged and the symptoms were continued till I was consulted in her case. The tumour was fifteen inches in circumference, covering the upper portion of the sacrum and four of the lumbar vertebræ, of a conical form, rising about seven inches, covered with integuments, and distended with fluid. She had managed by adjusting her clothes so as to keep the deformity from being much noticed. While riding in a carriage she was injured and put in pain by having the tumour pressed against the seats, and always afterwards suffered with the symptoms mentioned. Walking or whatever exercise fatigued her much, uniformly produced lassitude and nervous excitement. She had suffered from the great enlargement and the concussion of the tumour for several months anterior to my seeing her, more than at any previous period. Indeed she was reduced to a state of constant danger and distress. I advised an operation, which was made by dividing the upper portion of the tumour with a scalpel about five inches, encircling the prominent portion of integuments within two elliptical incisions. Twenty-two ounces of fluid were discharged, carrying the appearance of the purest alcohol. On standing till cold, it had no appearance of serum or lymph, but was more like distilled water. These incisions, and the removal of parts, exposed the interior and bottom of the tumour. It was lined with the dura mater distended and enlarged with the fluid mentioned, two of the lower spinous processes were wanting; a cavity was presented, and the spinal cord, in its natural position, and covered with the pia mater, was perceptible.

The divided parts were brought together by adhesive strips, and compresses being laid on a bandage was applied. She suffered but little by the operation, except the former symptoms from concussion; these were great for a few minutes. There was a constant secretion and discharge of fluid, from the parts for the first five days, with neuralgic symptoms, and increased arterial excitement, which were mitigated by large doses of morphia and warm fomentations, over the spine. The suppurative process took place freely about the tenth day, and a union of the parts followed, with a mitigation of all the symptoms. A sound state of the parts, with a depression of the integuments over the deranged portion of the spine, followed, and she has enjoyed good health up to this time, which is eight years. This tumour carried with it from the birth of the individual the appearance of those which I have described as curable. If my conclusions are correct, these "cysts," as "D." terms them, have secreting surfaces, and would not be likely to unite with a coagulum, or fibrine, deposited within the sac. Would the coagulum adhere, without inflammation, to the surrounding tissues?

*Painsville*, Ohio, July, 1840.

